# Supplementary material for: Exploring Core Genes by Comparative Transcriptomics Analysis for Early Diagnosis, Prognosis, and Therapies of Colorectal Cancer
Source: Cancers (Basel). 2023 Feb 21;15(5):1369. doi: 10.3390/cancers15051369 (PMC10000172; doi:10.3390/cancers15051369)
Supplement: Supplementary file 1 [file cancers-15-01369-s001.zip › cancers-2133624-supplementary.pdf]

Supplementary File

Exploring core genes by comparative transcriptomics analysis for early diagnosis, prognosis and therapies of colorectal-cancer

Md. Ariful Islam[1], Md. Bayazid Hossen[1], Md. Abu Horaira[1], Md Alim Hossen[1], Md. Kaderi Kibria[1], Md. Selim Reza[1], Khanis Farhana Tuly[1], Md. Omar Faruqe[2], Firoz Kabir[3], Rashidul Alam Mahumud[4*] and Md. Nurul Haque Mollah[1*]

[1] Bioinformatics Lab, Department of Statistics, University of Rajshahi, Rajshahi-6205, Bangladesh

[2] Department of Computer Science and Engineering, University of Rajshahi, Rajshahi-6205, Bangladesh

[3] Department of Ophthalmology and Visual Sciences, School of Medicine, University of Maryland, Baltimore, MD 21201, USA

[4] NHMRC Clinical Trials Centre, Faculty of Medicine and Health, The University of Sydney, Camperdown, NSW 2006, Australia

***Corresponding Author:** Md. Nurul Haque Mollah (E-mail: mollah.stat.bio@ru.ac.bd)

Tables/Figures	Captions/Legends	Pages
**Figure S1**	Box plots for the expressions of KGs with different stages (Stage 1, Stage 2, Stage 3, and Stage 4) of READ (Rectal adenocarcinoma) including the control stage in TCGA database.	2
**Figure S2**	Core genes-set enrichment analysis with different diseases	3
**Figure S3**	Pan-cancer analysis of core genes (A) AURKA, (B) CDC20, (C) CDK1, (D) CDKN3, (E) CKS2, (F) MAD2L1, (G) MELK, (H) PTTG1, (I) TOP2A, (J) TPX2	4-7
**Table S1**	Transcriptome-guided 158 meta-drug agents associated with CRC infections collected from DSigDB online database.	8
**Table S2**	The list of top 20 significantly (*p*-value<0.05) comorbidities associated with CGs	9
**Reference**		9

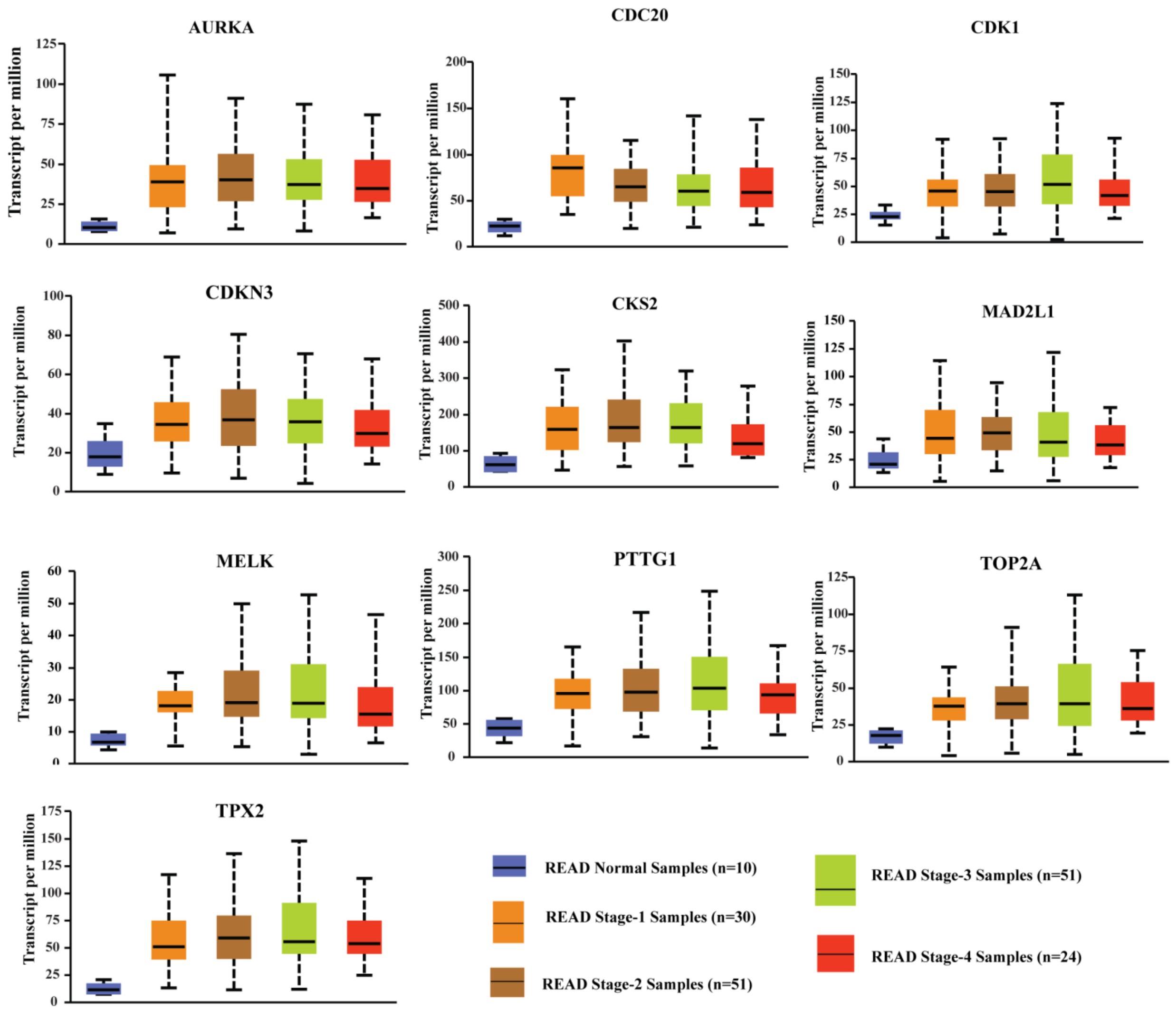

Figure S1. Box plots for the expressions of KGs with different stages (Stage 1, Stage 2, Stage 3, and Stage 4) of READ (Rectal adenocarcinoma) including the control stage in TCGA database.

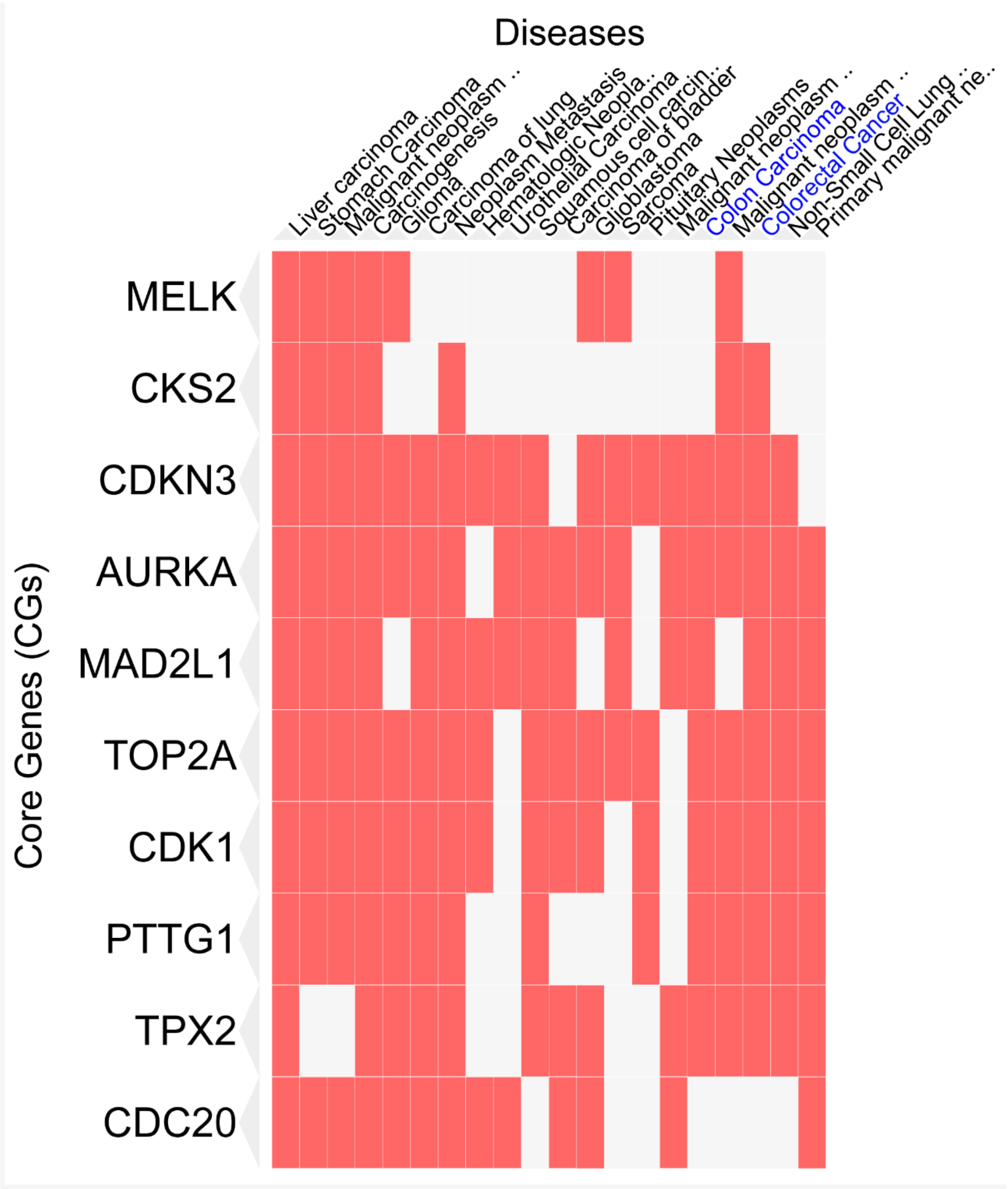

Figure S2. Core genes-set enrichment analysis with different diseases

(A)

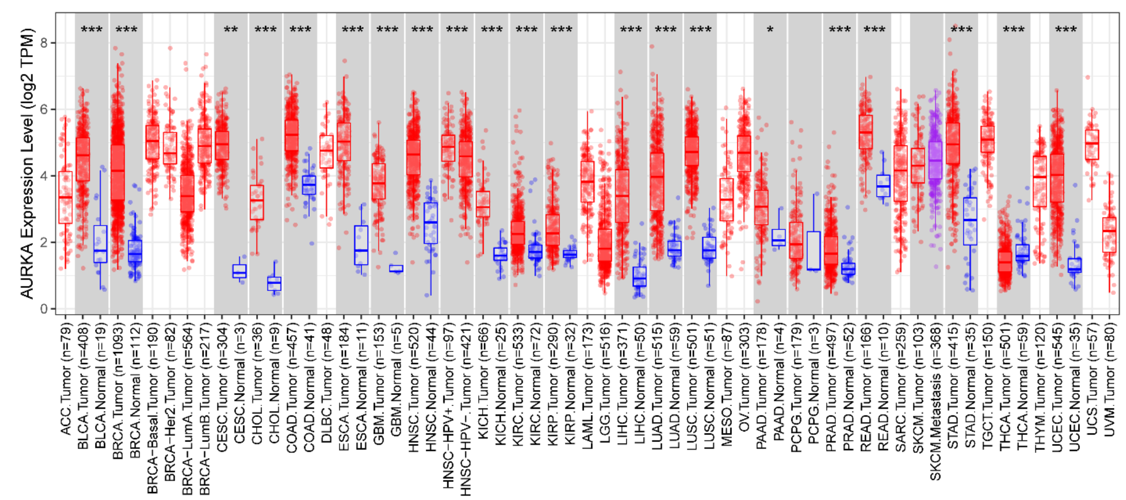
AURKA Expression Level (log2 TPM)
ACC.Tumor (n=79)
BLCA.Tumor (n=408)
BLCA.Normal (n=19)
BRCA.Tumor (n=1093)
BRCA.Normal (n=112)
BRCA−Basal.Tumor (n=190)
BRCA−Her2.Tumor (n=82)
BRCA−LumA.Tumor (n=564)
BRCA−LumB.Tumor (n=217)
CESC.Tumor (n=304)
CESC.Normal (n=3)
CHOL.Tumor (n=36)
CHOL.Normal (n=9)
COAD.Tumor (n=457)
COAD.Normal (n=41)
DLBC.Tumor (n=48)
ESCA.Tumor (n=184)
ESCA.Normal (n=11)
GBM.Tumor (n=153)
GBM.Normal (n=5)
HNSC.Tumor (n=520)
HNSC.Normal (n=44)
HNSC−HPV+.Tumor (n=97)
HNSC−HPV−.Tumor (n=421)
KICH.Tumor (n=66)
KICH.Normal (n=25)
KIRC.Tumor (n=533)
KIRC.Normal (n=72)
KIRP.Tumor (n=290)
KIRP.Normal (n=32)
LAML.Tumor (n=173)
LGG.Tumor (n=516)
LIHC.Tumor (n=371)
LIHC.Normal (n=50)
LUAD.Tumor (n=515)
LUAD.Normal (n=59)
LUSC.Tumor (n=501)
LUSC.Normal (n=51)
MESO.Tumor (n=87)
OV.Tumor (n=303)
PAAD.Tumor (n=178)
PAAD.Normal (n=4)
PCPG.Tumor (n=179)
PCPG.Normal (n=3)
PRAD.Tumor (n=497)
PRAD.Normal (n=52)
READ.Tumor (n=166)
READ.Normal (n=10)
SARC.Tumor (n=259)
SKCM.Tumor (n=103)
SKCM.Metastasis (n=368)
STAD.Tumor (n=415)
STAD.Normal (n=35)
TGCT.Tumor (n=150)
THCA.Tumor (n=501)
THCA.Normal (n=59)
THYM.Tumor (n=120)
UCEC.Tumor (n=545)
UCEC.Normal (n=35)
UCS.Tumor (n=57)
UVM.Tumor (n=80)

(B)

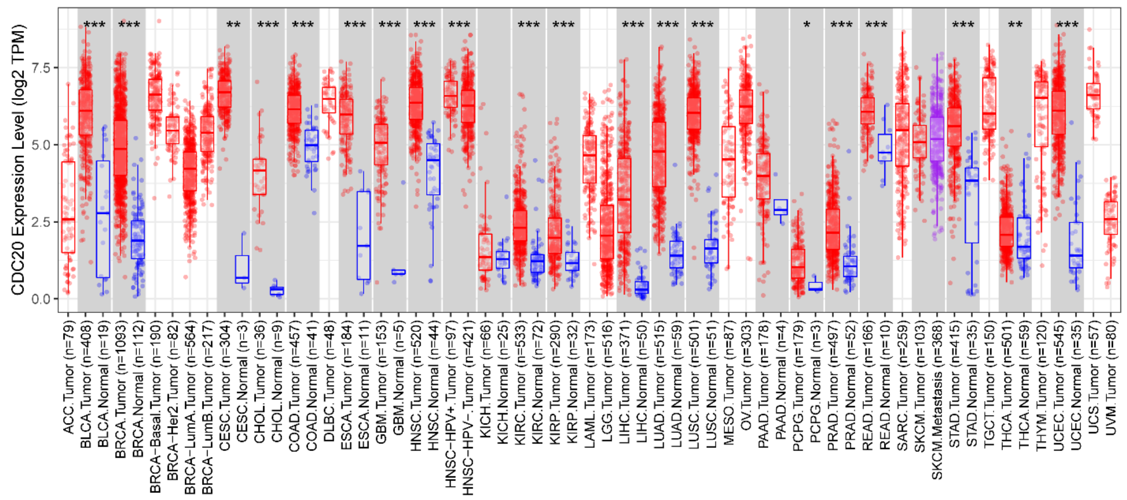
CDC20 Expression Level (log2 TPM)

(C)

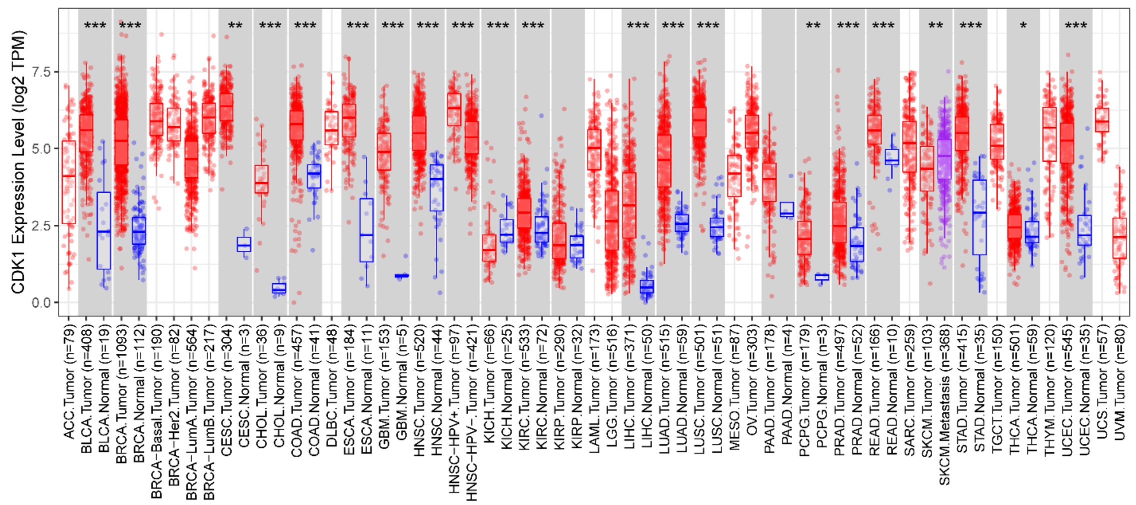
CDK1 Expression Level (log2 TPM)

(D)

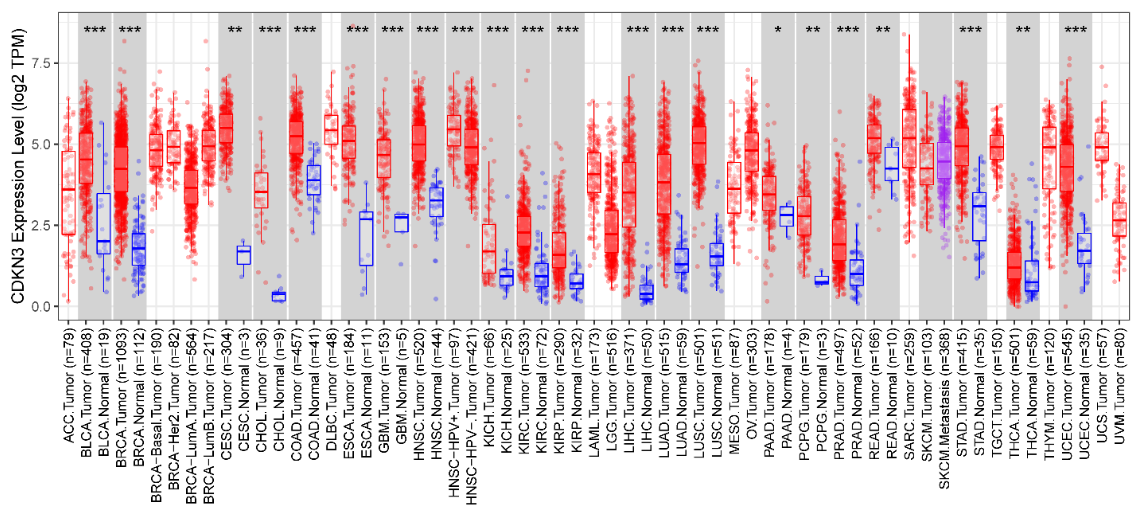
CDKN3 Expression Level (log2 TPM)
7.5
5.0
2.5
0.0
ACC.Tumor (n=79)
BLCA.Tumor (n=408)
BLCA.Normal (n=19)
BRCA.Tumor (n=1093)
BRCA.Normal (n=112)
BRCA−Basal.Tumor (n=190)
BRCA−Her2.Tumor (n=82)
BRCA−LumA.Tumor (n=564)
BRCA−LumB.Tumor (n=217)
CESC.Tumor (n=304)
CESC.Normal (n=3)
CHOL.Tumor (n=36)
CHOL.Normal (n=9)
COAD.Tumor (n=457)
COAD.Normal (n=41)
DLBC.Tumor (n=48)
ESCA.Tumor (n=184)
ESCA.Normal (n=11)
GBM.Tumor (n=153)
GBM.Normal (n=5)
HNSC.Tumor (n=520)
HNSC.Normal (n=44)
HNSC−HPV+.Tumor (n=97)
HNSC−HPV−.Tumor (n=421)
KICH.Tumor (n=66)
KICH.Normal (n=25)
KIRC.Tumor (n=533)
KIRC.Normal (n=72)
KIRP.Tumor (n=290)
KIRP.Normal (n=32)
LAML.Tumor (n=173)
LGG.Tumor (n=516)
LIHC.Tumor (n=371)
LIHC.Normal (n=50)
LUAD.Tumor (n=515)
LUAD.Normal (n=59)
LUSC.Tumor (n=501)
LUSC.Normal (n=51)
MESO.Tumor (n=87)
OV.Tumor (n=303)
PAAD.Tumor (n=178)
PAAD.Normal (n=4)
PCPG.Tumor (n=179)
PCPG.Normal (n=3)
PRAD.Tumor (n=497)
PRAD.Normal (n=52)
READ.Tumor (n=166)
READ.Normal (n=10)
SARC.Tumor (n=259)
SKCM.Tumor (n=103)
SKCM.Metastasis (n=368)
STAD.Tumor (n=415)
STAD.Normal (n=35)
TGCT.Tumor (n=150)
THCA.Tumor (n=501)
THCA.Normal (n=59)
THYM.Tumor (n=120)
UCEC.Tumor (n=545)
UCEC.Normal (n=35)
UCS.Tumor (n=57)
UVM.Tumor (n=80)

(E)

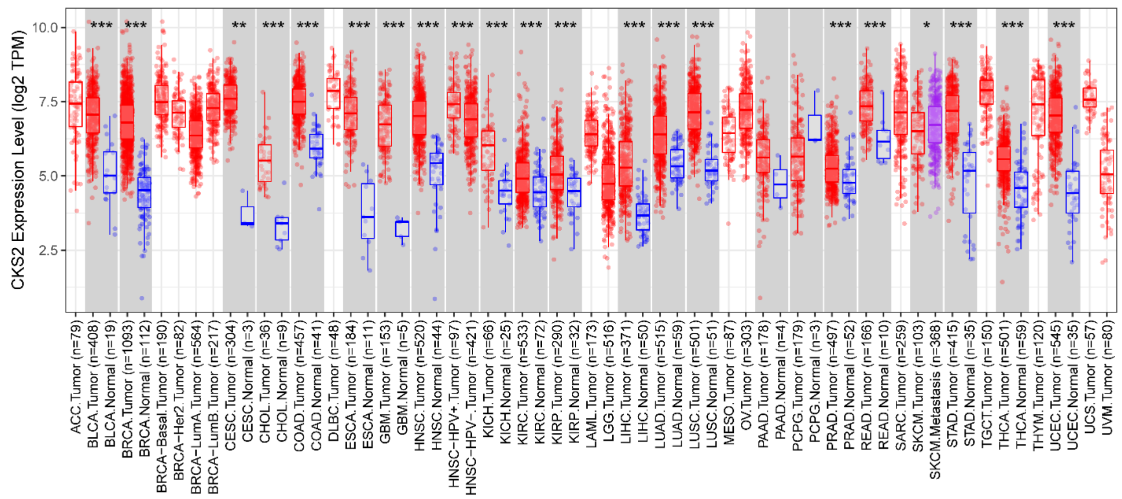
CKS2 Expression Level (log2 TPM)
10.0
7.5
5.0
2.5
ACC.Tumor (n=79)
BLCA.Tumor (n=408)
BLCA.Normal (n=19)
BRCA.Tumor (n=1093)
BRCA.Normal (n=112)
BRCA−Basal.Tumor (n=190)
BRCA−Her2.Tumor (n=82)
BRCA−LumA.Tumor (n=564)
BRCA−LumB.Tumor (n=217)
CESC.Tumor (n=304)
CESC.Normal (n=3)
CHOL.Tumor (n=36)
CHOL.Normal (n=9)
COAD.Tumor (n=457)
COAD.Normal (n=41)
DLBC.Tumor (n=48)
ESCA.Tumor (n=184)
ESCA.Normal (n=11)
GBM.Tumor (n=153)
GBM.Normal (n=5)
HNSC.Tumor (n=520)
HNSC.Normal (n=44)
HNSC−HPV+.Tumor (n=97)
HNSC−HPV−.Tumor (n=421)
KICH.Tumor (n=66)
KICH.Normal (n=25)
KIRC.Tumor (n=533)
KIRC.Normal (n=72)
KIRP.Tumor (n=290)
KIRP.Normal (n=32)
LAML.Tumor (n=173)
LGG.Tumor (n=516)
LIHC.Tumor (n=371)
LIHC.Normal (n=50)
LUAD.Tumor (n=515)
LUAD.Normal (n=59)
LUSC.Tumor (n=501)
LUSC.Normal (n=51)
MESO.Tumor (n=87)
OV.Tumor (n=303)
PAAD.Tumor (n=178)
PAAD.Normal (n=4)
PCPG.Tumor (n=179)
PCPG.Normal (n=3)
PRAD.Tumor (n=497)
PRAD.Normal (n=52)
READ.Tumor (n=166)
READ.Normal (n=10)
SARC.Tumor (n=259)
SKCM.Tumor (n=103)
SKCM.Metastasis (n=368)
STAD.Tumor (n=415)
STAD.Normal (n=35)
TGCT.Tumor (n=150)
THCA.Tumor (n=501)
THCA.Normal (n=59)
THYM.Tumor (n=120)
UCEC.Tumor (n=545)
UCEC.Normal (n=35)
UCS.Tumor (n=57)
UVM.Tumor (n=80)

(F)

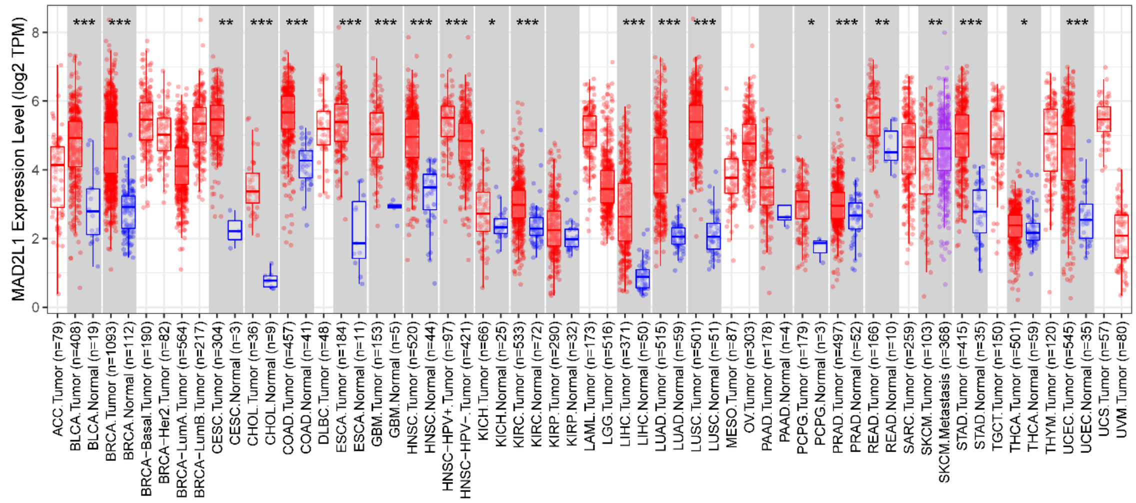
MAD2L1 Expression Level (log2 TPM)
8
6
4
2
0
ACC.Tumor (n=79)
BLCA.Tumor (n=408)
BLCA.Normal (n=19)
BRCA.Tumor (n=1093)
BRCA.Normal (n=112)
BRCA−Basal.Tumor (n=190)
BRCA−Her2.Tumor (n=82)
BRCA−LumA.Tumor (n=564)
BRCA−LumB.Tumor (n=217)
CESC.Tumor (n=304)
CESC.Normal (n=3)
CHOL.Tumor (n=36)
CHOL.Normal (n=9)
COAD.Tumor (n=457)
COAD.Normal (n=41)
DLBC.Tumor (n=48)
ESCA.Tumor (n=184)
ESCA.Normal (n=11)
GBM.Tumor (n=153)
GBM.Normal (n=5)
HNSC.Tumor (n=520)
HNSC.Normal (n=44)
HNSC−HPV+.Tumor (n=97)
HNSC−HPV−.Tumor (n=421)
KICH.Tumor (n=66)
KICH.Normal (n=25)
KIRC.Tumor (n=533)
KIRC.Normal (n=72)
KIRP.Tumor (n=290)
KIRP.Normal (n=32)
LAML.Tumor (n=173)
LGG.Tumor (n=516)
LIHC.Tumor (n=371)
LIHC.Normal (n=50)
LUAD.Tumor (n=515)
LUAD.Normal (n=59)
LUSC.Tumor (n=501)
LUSC.Normal (n=51)
MESO.Tumor (n=87)
OV.Tumor (n=303)
PAAD.Tumor (n=178)
PAAD.Normal (n=4)
PCPG.Tumor (n=179)
PCPG.Normal (n=3)
PRAD.Tumor (n=497)
PRAD.Normal (n=52)
READ.Tumor (n=166)
READ.Normal (n=10)
SARC.Tumor (n=259)
SKCM.Tumor (n=103)
SKCM.Metastasis (n=368)
STAD.Tumor (n=415)
STAD.Normal (n=35)
TGCT.Tumor (n=150)
THCA.Tumor (n=501)
THCA.Normal (n=59)
THYM.Tumor (n=120)
UCEC.Tumor (n=545)
UCEC.Normal (n=35)
UCS.Tumor (n=57)
UVM.Tumor (n=80)

(G)

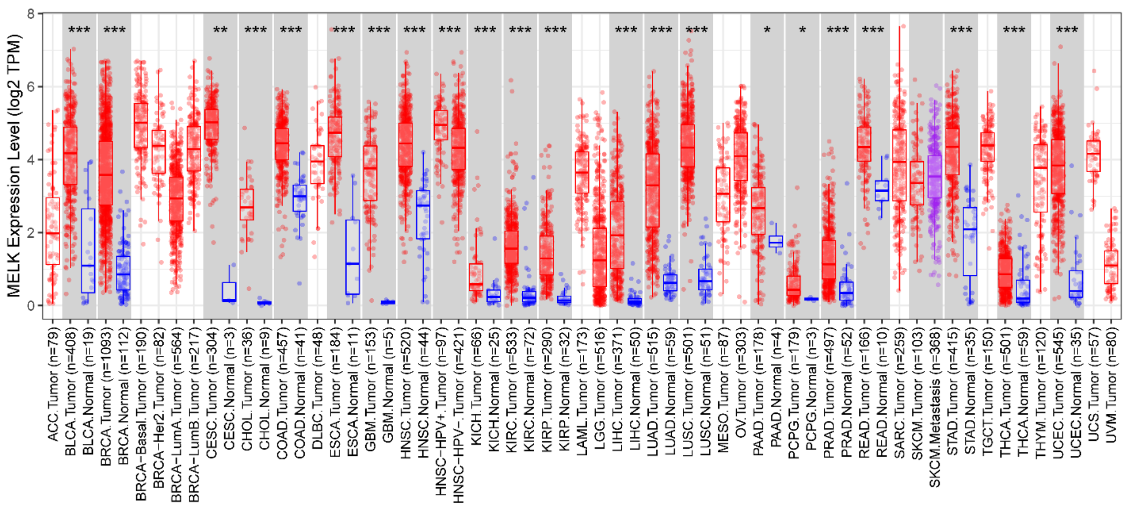
MELK Expression Level (log2 TPM)
0
2
4
6
8
ACC.Tumor (n=79)
BLCA.Tumor (n=408)
BLCA.Normal (n=19)
BRCA.Tumor (n=1093)
BRCA.Normal (n=112)
BRCA−Basal.Tumor (n=190)
BRCA−Her2.Tumor (n=82)
BRCA−LumA.Tumor (n=564)
BRCA−LumB.Tumor (n=217)
CESC.Tumor (n=304)
CESC.Normal (n=3)
CHOL.Tumor (n=36)
CHOL.Normal (n=9)
COAD.Tumor (n=457)
COAD.Normal (n=41)
DLBC.Tumor (n=48)
ESCA.Tumor (n=184)
ESCA.Normal (n=11)
GBM.Tumor (n=153)
GBM.Normal (n=5)
HNSC.Tumor (n=520)
HNSC.Normal (n=44)
HNSC−HPV+.Tumor (n=97)
HNSC−HPV−.Tumor (n=421)
KICH.Tumor (n=66)
KICH.Normal (n=25)
KIRC.Tumor (n=533)
KIRC.Normal (n=72)
KIRP.Tumor (n=290)
KIRP.Normal (n=32)
LAML.Tumor (n=173)
LGG.Tumor (n=516)
LIHC.Tumor (n=371)
LIHC.Normal (n=50)
LUAD.Tumor (n=515)
LUAD.Normal (n=59)
LUSC.Tumor (n=501)
LUSC.Normal (n=51)
MESO.Tumor (n=87)
OV.Tumor (n=303)
PAAD.Tumor (n=178)
PAAD.Normal (n=4)
PCPG.Tumor (n=179)
PCPG.Normal (n=3)
PRAD.Tumor (n=497)
PRAD.Normal (n=52)
READ.Tumor (n=166)
READ.Normal (n=10)
SARC.Tumor (n=259)
SKCM.Tumor (n=103)
SKCM.Metastasis (n=368)
STAD.Tumor (n=415)
STAD.Normal (n=35)
TGCT.Tumor (n=150)
THCA.Tumor (n=501)
THCA.Normal (n=59)
THYM.Tumor (n=120)
UCEC.Tumor (n=545)
UCEC.Normal (n=35)
UCS.Tumor (n=57)
UVM.Tumor (n=80)

(H)

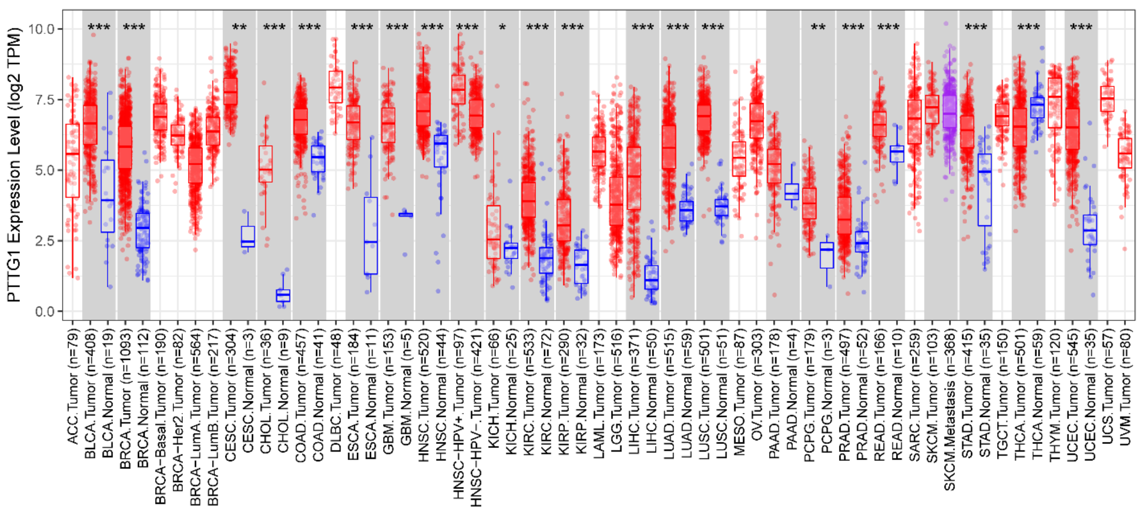
PTTG1 Expression Level (log2 TPM)
0.0
2.5
5.0
7.5
10.0
ACC.Tumor (n=79)
BLCA.Tumor (n=408)
BLCA.Normal (n=19)
BRCA.Tumor (n=1093)
BRCA.Normal (n=112)
BRCA−Basal.Tumor (n=190)
BRCA−Her2.Tumor (n=82)
BRCA−LumA.Tumor (n=564)
BRCA−LumB.Tumor (n=217)
CESC.Tumor (n=304)
CESC.Normal (n=3)
CHOL.Tumor (n=36)
CHOL.Normal (n=9)
COAD.Tumor (n=457)
COAD.Normal (n=41)
DLBC.Tumor (n=48)
ESCA.Tumor (n=184)
ESCA.Normal (n=11)
GBM.Tumor (n=153)
GBM.Normal (n=5)
HNSC.Tumor (n=520)
HNSC.Normal (n=44)
HNSC−HPV+.Tumor (n=97)
HNSC−HPV−.Tumor (n=421)
KICH.Tumor (n=66)
KICH.Normal (n=25)
KIRC.Tumor (n=533)
KIRC.Normal (n=72)
KIRP.Tumor (n=290)
KIRP.Normal (n=32)
LAML.Tumor (n=173)
LGG.Tumor (n=516)
LIHC.Tumor (n=371)
LIHC.Normal (n=50)
LUAD.Tumor (n=515)
LUAD.Normal (n=59)
LUSC.Tumor (n=501)
LUSC.Normal (n=51)
MESO.Tumor (n=87)
OV.Tumor (n=303)
PAAD.Tumor (n=178)
PAAD.Normal (n=4)
PCPG.Tumor (n=179)
PCPG.Normal (n=3)
PRAD.Tumor (n=497)
PRAD.Normal (n=52)
READ.Tumor (n=166)
READ.Normal (n=10)
SARC.Tumor (n=259)
SKCM.Tumor (n=103)
SKCM.Metastasis (n=368)
STAD.Tumor (n=415)
STAD.Normal (n=35)
TGCT.Tumor (n=150)
THCA.Tumor (n=501)
THCA.Normal (n=59)
THYM.Tumor (n=120)
UCEC.Tumor (n=545)
UCEC.Normal (n=35)
UCS.Tumor (n=57)
UVM.Tumor (n=80)

(I)

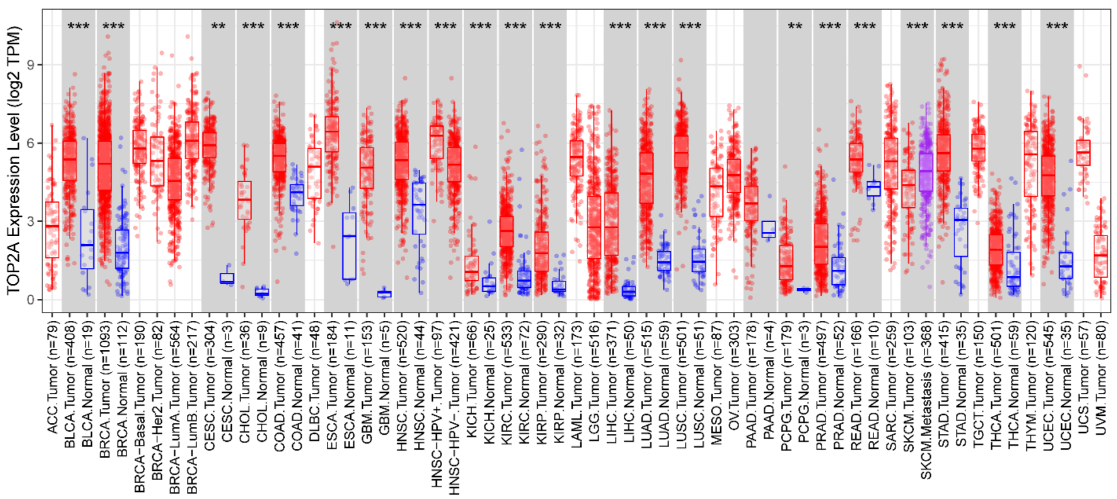
TOP2A Expression Level (log2 TPM)
0
3
6
9
ACC.Tumor (n=79)
BLCA.Tumor (n=408)
BLCA.Normal (n=19)
BRCA.Tumor (n=1093)
BRCA.Normal (n=112)
BRCA−Basal.Tumor (n=190)
BRCA−Her2.Tumor (n=82)
BRCA−LumA.Tumor (n=564)
BRCA−LumB.Tumor (n=217)
CESC.Tumor (n=304)
CESC.Normal (n=3)
CHOL.Tumor (n=36)
CHOL.Normal (n=9)
COAD.Tumor (n=457)
COAD.Normal (n=41)
DLBC.Tumor (n=48)
ESCA.Tumor (n=184)
ESCA.Normal (n=11)
GBM.Tumor (n=153)
GBM.Normal (n=5)
HNSC.Tumor (n=520)
HNSC.Normal (n=44)
HNSC−HPV+.Tumor (n=97)
HNSC−HPV−.Tumor (n=421)
KICH.Tumor (n=66)
KICH.Normal (n=25)
KIRC.Tumor (n=533)
KIRC.Normal (n=72)
KIRP.Tumor (n=290)
KIRP.Normal (n=32)
LAML.Tumor (n=173)
LGG.Tumor (n=516)
LIHC.Tumor (n=371)
LIHC.Normal (n=50)
LUAD.Tumor (n=515)
LUAD.Normal (n=59)
LUSC.Tumor (n=501)
LUSC.Normal (n=51)
MESO.Tumor (n=87)
OV.Tumor (n=303)
PAAD.Tumor (n=178)
PAAD.Normal (n=4)
PCPG.Tumor (n=179)
PCPG.Normal (n=3)
PRAD.Tumor (n=497)
PRAD.Normal (n=52)
READ.Tumor (n=166)
READ.Normal (n=10)
SARC.Tumor (n=259)
SKCM.Tumor (n=103)
SKCM.Metastasis (n=368)
STAD.Tumor (n=415)
STAD.Normal (n=35)
TGCT.Tumor (n=150)
THCA.Tumor (n=501)
THCA.Normal (n=59)
THYM.Tumor (n=120)
UCEC.Tumor (n=545)
UCEC.Normal (n=35)
UCS.Tumor (n=57)
UVM.Tumor (n=80)

(J)

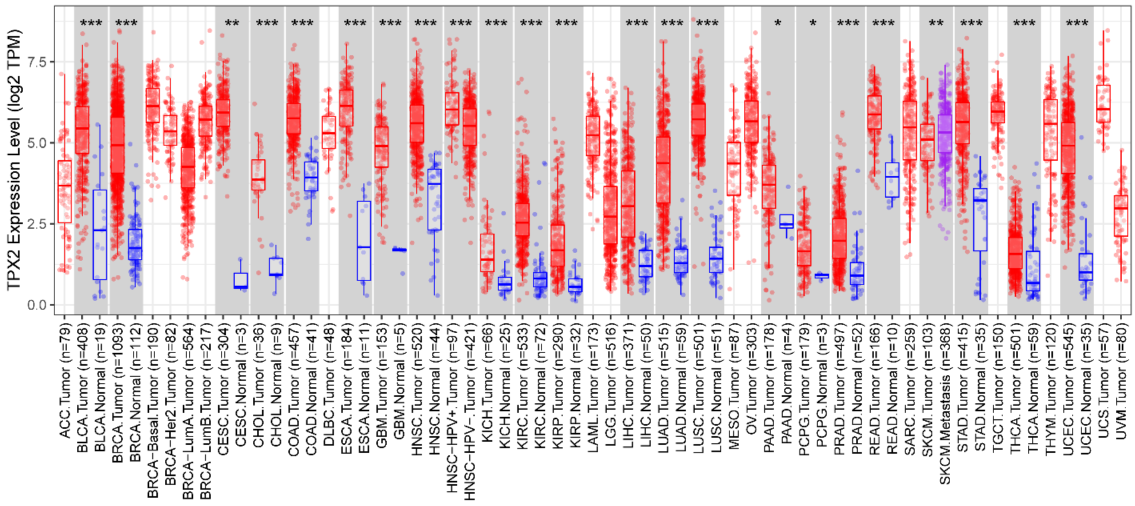

Figure S3: Pan-cancer analysis of core genes (A) AURKA, (B) CDC20, (C) CDK1, (D) CDKN3, (E) CKS2, (F) MAD2L1, (G) MELK, (H) PTTG1, (I) TOP2A, (J) TPX2

Table S1: Transcriptome-guided 158 meta-drug agents associated with CRC infections collected from DSigDB online database.

Source	Drug
DSigDB[1]	Manzamine A, Cardidigin, Staurosporine, Sitosterol, Benzo.a.pyrene, Nocardiopsis Sp, Riccardin D, Troglitazone, Irinotecan Hydrochloride, Masitinib, Peiminine, Irinotecan, Maslinic Acid, Etoposide, Nortopsentin A, Epigallocatechin Gallate, Procyanidin, Regorafenib, Foretinib, Simvastatin, Hederagenin, AZD4547, Quercitrin, JAK 3 Inhibitor, Caulerpin, AFLATOXIN, Doxorubicin, Ellipticine, Paclitaxel, Calcitriol, COUMESTROL, Lobelin, Luteolin, Methotrexate, Calcium.Folinate, Enterolactone, Peridinin, Cdk.12.Inhibitor, Icariin, Astaxanthin, OTSSP167, STL264925, CP466722, Dasatinib, Quercetin, Nintedanib, SB 202190, Bicalutamide, Belinostat, Indirubin Derivative, Sipholenol L, Chlortetracycline, Sipholenol A, Vinblastine, CHEMBL35349, Capecitabine, Deoxyelephantopin, Fulvestrant, Milnamide D, Candidaspongiolide B, IPA3, Physcion, Docetaxel, Thapsigargin, Estradiol, Genistein, Binimetinib, Piroxicam, Thalidomide, Sipholenone A, Yuanhuacine, Sexangularetin, Cobimetinib, Danazol, Fucoxanthin, PKR Inhibitor, Andrographolide, Progesterone, Gambogic Acid, Curcumin, Violaxanthin, Resveratrol, Trifluridine, Kahweol, Curcumin, Testosterone, Encorafenib, Trichostatin A, Indigocarpan, Resveratrol, Trifluridine, Cyclosporin A, Goniothalamin, Gingerol, BI D1870, Candidaspongiolide A, Renieramycin M, Cardamonin, Candidaspongiolide Core, PhIP, Gliotoxin, Dronabinol, Gefitinib, Leptomycin, BX795, MG132, Bisphenol.A, Monobenzone, Oxymatrine, Isoledene, Retinoic.Acid, Okadaic.Acid, Salternamide.A, Ciclopirox, LUCANTHONE, Hyoscyamine, ZINC214489228, MeIQx, Clindamycin, Zerumbone, Esculetin, Deferoxamine, Tripolinolate A, Thioguanosine, Fluorouracil.Deoxyriboside, Vorinostat, Thymoquinone, Uracil, Fluorouracil Mononitrate, Mertensene, Levamisole, Phorbol 12 Myristate 13 Acetate, Methylcholine, Dmnq, Cuminaldehyde, Fluorouracil, Diaminocyclohexane, Sulforaphane, Methyl.Ferulate, Tegafur, Azacytidine, Tipiracil, Dihydrouracil, Pyrazolo 34D Pyrimidine, Dihydrotegafur, Pentanone, Cardol, Oplopantriol A, Ethoxy 5 Fluorouracil, Bromoisatin, Oxalic Acid, Cadmium Acetate, Decitabine, Fucoidan.Alaria, Hydrogen.Peroxide, MERCURY, Nickel, Tetrachloroethylene.

Table S2: The list of top 20 significantly (p-value<0.05) comorbidities associated with CGs

Disease	Adjusted *P*-value	Core genes (CGs)
Liver carcinoma	5.86E-06	TOP2A;CDC20;TPX2;MELK;PTTG1;CDK1;CKS2;AURKA;CDKN3;MAD2L1
Stomach Carcinoma	5.86E-06	TOP2A;CDC20;MELK;PTTG1;CDK1;CKS2;AURKA;CDKN3;MAD2L1
Malignant neoplasm of stomach	5.86E-06	TOP2A;CDC20;MELK;PTTG1;CDK1;CKS2;AURKA;CDKN3;MAD2L1
Carcinogenesis	1.16E-05	TOP2A;CDC20;TPX2;MELK;PTTG1;CDK1;CKS2;AURKA;CDKN3;MAD2L1
Glioma	6.30E-05	TOP2A;CDC20;TPX2;MELK;PTTG1;CDK1;AURKA;CDKN3
Carcinoma of lung	1.27E-04	TOP2A;CDC20;TPX2;PTTG1;CDK1;AURKA;CDKN3;MAD2L1
Neoplasm Metastasis	1.80E-04	TOP2A;CDC20;TPX2;PTTG1;CDK1;CKS2;AURKA;CDKN3;MAD2L1
Hematologic Neoplasms	1.80E-04	TOP2A;CDC20;CDK1;CDKN3;MAD2L1
Urothelial Carcinoma	2.28E-04	CDC20;AURKA;CDKN3;MAD2L1
Squamous cell carcinoma	2.29E-04	TOP2A;TPX2;PTTG1;CDK1;AURKA;CDKN3;MAD2L1
Carcinoma of bladder	2.31E-04	TOP2A;CDC20;TPX2;CDK1;AURKA;MAD2L1
Glioblastoma	2.37E-04	TOP2A;CDC20;TPX2;MELK;CDK1;AURKA;CDKN3
Sarcoma	2.42E-04	TOP2A;MELK;AURKA;CDKN3;MAD2L1
Pituitary Neoplasms	2.42E-04	TOP2A;PTTG1;CDK1;CDKN3
Malignant neoplasm of esophagus	2.42E-04	CDC20;TPX2;AURKA;CDKN3;MAD2L1
Colon Carcinoma	2.97E-04	TOP2A;TPX2;PTTG1;CDK1;AURKA;CDKN3;MAD2L1
Malignant neoplasm of prostate	3.56E-04	TOP2A;TPX2;MELK;PTTG1;CDK1;CKS2;AURKA;CDKN3
Colorectal Cancer	3.86E-04	TOP2A;TPX2;PTTG1;CDK1;CKS2;AURKA;CDKN3;MAD2L1
Non-Small Cell Lung Carcinoma	4.00E-04	TOP2A;TPX2;PTTG1;CDK1;AURKA;CDKN3;MAD2L1
Primary malignant neoplasm of lung	4.10E-04	TOP2A;CDC20;TPX2;PTTG1;CDK1;AURKA;MAD2L1

References

1. Yoo M, Shin J, Kim J, Ryall KA, Lee K, Lee S, et al. DSigDB: Drug signatures database for gene set analysis. Bioinformatics. 2015;31.
